# Supplementary material for: A tool for identifying green solvents for printed electronics
Source: Nat Commun. 2021 Jul 23;12:4510. doi: 10.1038/s41467-021-24761-x (PMC8302666; doi:10.1038/s41467-021-24761-x)
Supplement: Supplementary file 1 — Supplementary Information [file 41467_2021_24761_MOESM1_ESM.pdf]

Supplementary information for

A Tool for Identifying Green Solvents for Printed Electronics

Christian Larsen,[1,2,†] Petter Lundberg,[1,†] Shi Tang,[1,2,†] Joan Ràfols-Ribé,[1] Andreas Sandström,[1,2] E. Mattias Lindh,[1] Jia Wang[1] and Ludvig Edman[1,2,*]

[1] The Organic Photonics and Electronics Group, Department of Physics, Umeå University, SE-90187 Umeå, Sweden

[2] LunaLEC AB, Linnaeus väg 24, SE-90187 Umeå, Sweden

[†] These authors contributed equally

[*] E-mail: ludvig.edman@umu.se

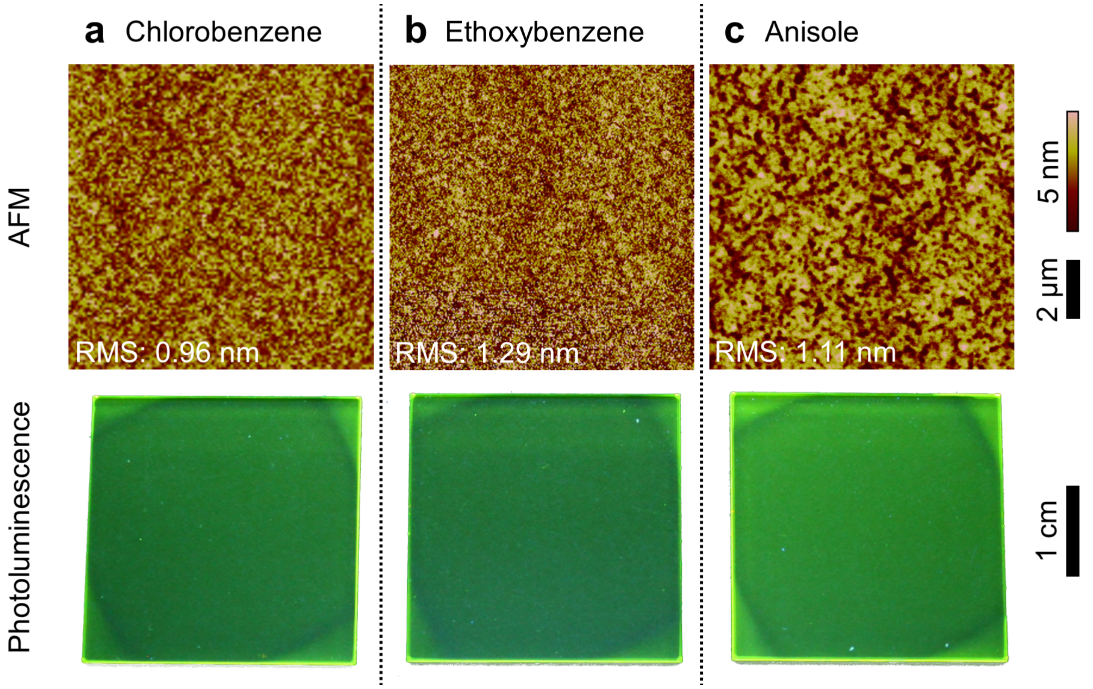

Figure S1. Investigation of uniformity of spin-coated active-material films. AFM images (top row) and photographs of the UV-activated photoluminescence (bottom row) from active-material films spin-coated from inks based on the solvent of **a** chlorobenzene, **b** ethoxybenzene, and **c** anisole. The low surface roughness in AFM and the uniform spatial emission in photoluminescence suggest that both microscopic and macroscopic film uniformity are obtained by spin coating the three inks.

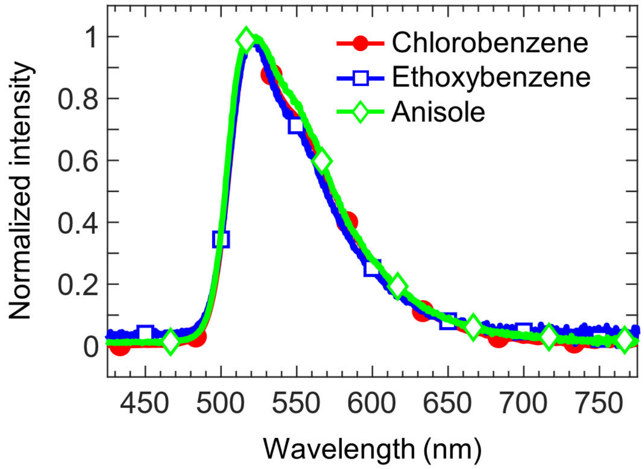

Figure S2. The influence of ink-solvent selection on the emission color. Normalized electroluminescence spectra of LEC devices fabricated by spin coating active-material inks based on chlorobenzene (solid red circles), ethoxybenzene (open blue squares) and anisole (open green diamonds). The essentially identical electroluminescence spectra reveal that the emission color is invariant to the selection of ink solvent.

Table S1. Summary of performance metrics for the spin-coated LECs. The device performance is presented as a function of ink-solvent selection and ink-storage time. All devices were driven by $j = 77$ A m^{-2}.

Ink solvent	Ink Storage	Turn-on time[a)] (s)	Peak luminance (cd m^{-2})	Min. voltage (V)	Current efficacy (cd A^{-1})	Power efficiency (lm W^{-1})	Lifetime[b)] (h)
Chlorobenzene	<1day	<2	3100	9.3	39.9	11.1	2.6
	30 days	4	2327	9.7	30.0	8.3	2.6
Ethoxybenzene	<1 day	<2	2530	9.3	32.6	9.2	2.9
	30 days	7	2434	8.6	31.7	9.4	2.8
Anisole	<1 day	<2	2556	9.1	33.2	9.7	2.5
	30 days	3	2292	8.7	29.8	8.2	3.3

a) Turn-on time to a luminance >1000 cd m^{-2}.
b) Lifetime at a luminance >1000 cd m^{-2}.

Note that the values for the peak luminance of 3100 cd m^{-2} and the current efficacy of 39.9 cd A^{-1} for the spin-coated LEC fabricated from the fresh chlorobenzene ink are slightly lower than the values reported in our previous publication[1] (3250 cd m^{-2} and $\eta = 42.2$ cd A^{-1}); we tentatively attribute this minor deviation to a batch-to-batch variation.

Table S2. Summary of performance metrics for the bar-coated LECs. The device performance is presented as a function of ink-solvent selection (using fresh inks). All devices were driven by $j = 77$ A m^{-2}.

Ink solvent	Turn-on time[a)] (s)	Peak luminance (cd m^{-2})	Min. voltage (V)	Current efficacy (cd A^{-1})	Power efficiency (lm W^{-1})	Lifetime[b)] (h)
Chlorobenzene	<2	1920	12.5	25.4	5.0	1.2
Ethoxybenzene	<2	2120	10.8	27.1	6.5	1.9
Anisole	<2	1680	11.8	22.0	5.0	1.4

a) Turn-on time to a luminance >1000 cd m^{-2}.
b) Lifetime at a luminance >1000 cd m^{-2}.

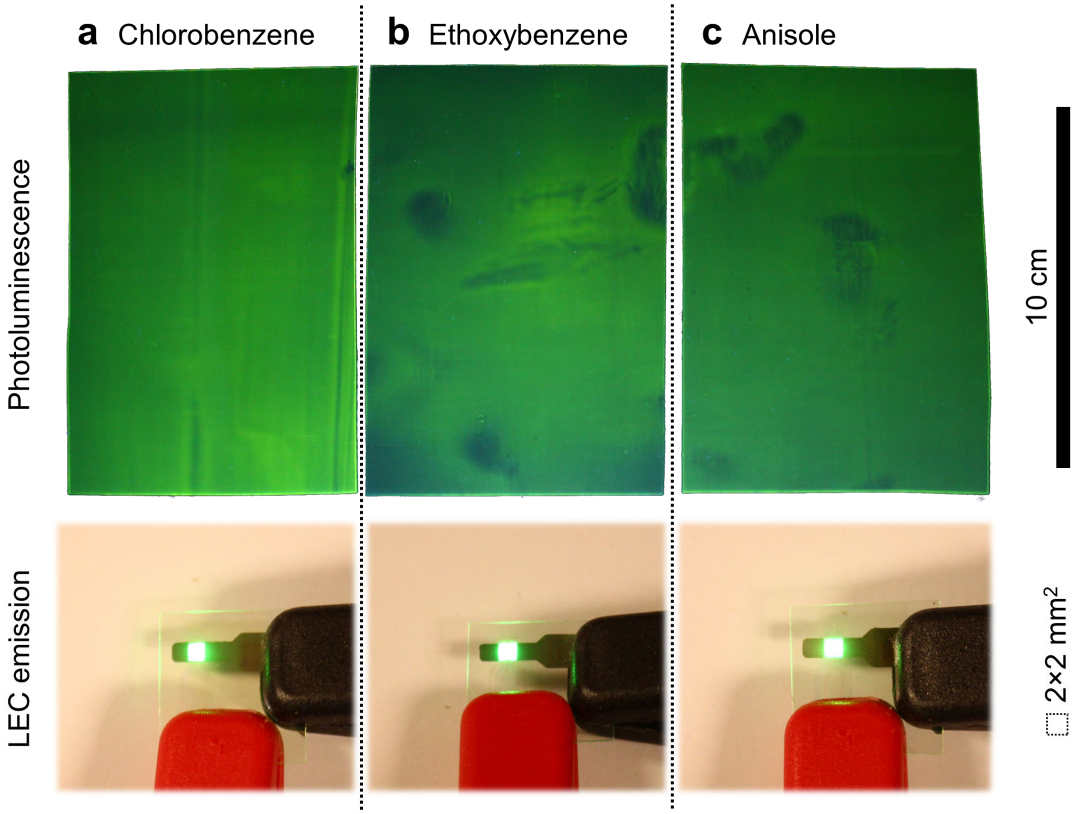

Figure S3. Investigation of the uniformity of the bar-coated active-material films and the corresponding LEC emission. Photographs of the UV-excited photoluminescence of bar-coated large-area active-material films (upper row) and the electroluminescence from bar-coated 2 × 2 mm² LEC devices (lower row) that were fabricated using inks based on **a** chlorobenzene, **b** ethoxybenzene, and **c** anisole. The photographs of the electroluminescence were captured after ~5 minutes operation using the following settings: ISO-800, F/5, 1/40 s. The spatial uniformity of both the photoluminescence (with the exception for some minor surface defects) and the electroluminescence demonstrates that uniform and pinhole-free films can be fabricated with bar-coating using all three ink solvents.

References

1 Tang, S. *et al.* Design rules for light-emitting electrochemical cells delivering bright luminance at 27.5 percent external quantum efficiency. *Nature communications* **8**, 1190, doi:10.1038/s41467-017-01339-0 (2017).
